# REC8 regulates neuroblastoma cell proliferation, migration, invasion, and angiogenesis via STAT3/VEGF signaling

**DOI:** 10.1186/s43046-023-00197-w

**Published:** 2023-12-18

**Authors:** Qiang Wang, Wei Fan, ZengHui Hao, Bingxue Liang, Meili Fan, Zijian Zhao, Zhaozhu Li

**Affiliations:** 1 Department of Pediatric Surgery, The Sixth Affiliated Hospital of Harbin Medical University, Harbin, 150001 China; 2 Department of Pediatric Surgery, The First Affiliated Hospital of Harbin Medical University, Harbin, 150001 China

**Keywords:** REC8, Neuroblastoma, Angiogenesis, Invasion, STAT3/VEGF

## Abstract

**Background:**

Neuroblastoma, one of the most prevalent childhood cancers, is often treated with surgery, radiation, and chemotherapy. However, prognosis and survival are still dismal for children with neuroblastoma at high risk. Consequently, it is vital to identify new and effective treatment targets. As a component of the meiotic cohesion complex, REC8 is involved in a wide range of malignancies. The current work assessed the impact of REC8 knockdown on SH-SY5Y and SK-N-AS neuroblastoma cells and delved into the molecular mechanism behind this effect.

**Methods:**

Knockdown of REC8 using the small interfering (si) RNA technology, and the results were verified by quantitative reverse transcriptase polymerase chain reaction (qRT-PCR) and western blot. The Cell Counting Kit-8 (CCK-8) was used to examine cell proliferation, while flow cytometry was used to examine cell cycle progression and apoptosis. Analyses of angiogenesis included tube formation experiments. Transwell tests were used to examine cell migration and invasion.

**Results:**

The data showed that downregulation of the REC8 led to a substantial decrease in cell proliferation by stopping the cell cycle in the G1 phase. REC8 knockdown significantly reduced neuroblastoma cell proliferation, migration, invasion, angiogenesis, induced cell cycle arrest, and enhanced apoptosis. We also discovered that repressing REC8 expression in neuroblastoma cell lines SH-SY5Y and SK-N-AS reduced their ability to activate the STAT3/VEGF signaling pathway.

**Conclusions:**

Neuroblastoma therapy may benefit from targeting REC8 and its downstream targets.

## Introduction

Neuroblastoma (NB), an embryonic malignancy derived from the neural crest, is a viral disease threatening children’s health, causing nearly 15% of deaths of children among pediatric malignancies [1, 2]. Despite aggressive multimodal therapy, NB is characterized by heterogeneous biological behaviors, including spontaneous regression or aggressive progression [3, 4]. No cure or therapy exists now that would provide patients with a favorable prognosis regardless of the stage of the disease [5]. To lessen the harmful side effects and decrease the dosage necessary for medication treatment, researchers should identify the abnormal signaling pathways in neuroblastoma and screen for potential inhibitors to substitute for traditional chemotherapy [6, 7]. Understanding the fundamental biological underpinnings of neuroblastoma is critical for developing novel approaches to improve prognostic stratification and patient outcomes.

REC8 is cohesin which helps keep chromosomes in good shape by performing structural maintenance chores [8]. REC8 is usually suppressed in mitotic proliferation, whose role in cancer has recently been subject to controversy [9–11]. Recent research has demonstrated that REC8 enhances stemness and promotes metastasis of colorectal cancer through BTK/Akt/β-catenin signaling pathway [12]. At the same time, Liu et al. found the expression of this gene to suppress tumor angiogenesis and progression in gastric cancer cells [13]. Unfortunately, neither the expression nor the function of REC8 in NB has been confirmed. Therefore, it was decided to study the effects of REC8 on the human neuroblastoma cell lines, specifically looking at how these processes relate to NB cell motility, invasion, and angiogenesis.

## Methods and materials

### Patients and tissues

In our study, 8 patients with primary neuroblastomas with a pathological diagnosis were selected from The Sixth Affiliated Hospital of Harbin Medical University. The fresh tissue samples were immediately snap-frozen in liquid nitrogen and stored at – 80 °C for western blotting experiments.

### Cell culture and transfection

Human NBL cell lines (BE (2)-C, IMR-32, SK-N-SH, and SH-SY5Y) and normal dorsal root ganglia (DG) cells were gained from the Chinese Academy of Sciences (Shanghai, China), which were cultured in an F12 medium (Gibco) and Eagle’s minimal essential medium (Gibco) supplemented with 10% heat-inactivated fetal bovine serum (Gemini). All cell lines were incubated at 37 °C in a humid incubator with air containing 5% CO_2_. The cells were transfected with siRNA (siREC8 and negative control siNC) using Lipofectamine™ RNAimax (Thermo Fisher) according to the manufacturer's instructions. Riobio Biotechnology synthesized siRNAs.

### Cell proliferation assays

Cells were seeded at 1 × 10^4^ cells/well in 96-well plates, and three multiple wells were set in each group. After 24 h of incubation, the old medium was discarded, and then the different treatments were added separately. After continued incubation for 24 h, 10 μL CCK⁃8 (Solarbio, China) was added, and the OD value at 450 nm was detected using a microplate reader after incubation for 4 h at 37 °C.

### Quantitative real-time PCR

TRIzol reagent (Invitrogen, USA) extracted total RNA from cells. Reverse transcription reactions were performed with a reverse transcription kit (Takara). SYBR Green Master Mix (Takara) was used for quantitative real-time PCR (qRT-PCR). The results were analyzed by the 2-ΔΔCt method.

### Western blot

Protein extraction and western blot were performed as previously described [14]. Antibodies were as follows: REC8 (1:1,000; Abcam), VEGF (1:1,000; CST), STAT3 (Ser-473; 1:1,000; CST), p-STAT3 (1:1,000; CST), β-Actin (1:5,000; Abcam), and GAPDH (1:5,000; Abcam).

### Colony formation assay

For 2 weeks, cells were grown after being seeded onto 6-well plates. After washing, fixing with 4% paraformaldehyde for 30 min, staining with 0.1% crystal violet for 1 min, and counting.

### Flow cytometry

The cells in each group were collected and washed in PBS, and 100 μl cells were mixed with 5 μl Annexin V-FITC and 5 μl Propidium iodide (PI) (Vazyme), then incubated in the dark for 15 min, and then the cell apoptosis was determined by flow cytometry.

### Transwell migration and invasion assay

Cell migration: Cells in each group were digested with trypsin, centrifuged, and resuspended with a serum-free medium at a density of 2.5 × 10^6^/ml. 200 μL of suspension was added to the upper chamber of Transwell, and 600 μL of culture medium containing 10 g/dL fetal bovine serum was added to the lower chamber, and the cells were cultured for 24 h. After fixation with formaldehyde and crystal violet staining, the number of transmembrane cells was observed under an inverted microscope. Cell invasion: The matrigel was diluted with a culture medium, spread flat on the top of the chamber, and air-dried. The other experimental procedures were the same as the detection of cell migration.

### Statistical analysis

Mean and standard deviation (SD) were used to illustrate the data. The two-sample Student’s *t* test was used to analyze the differences between the two groups. Statistical significance was assumed at the 0.05 level or below.

## Results

### REC8 expression was upregulated in NB cells

**Fig. 1:**
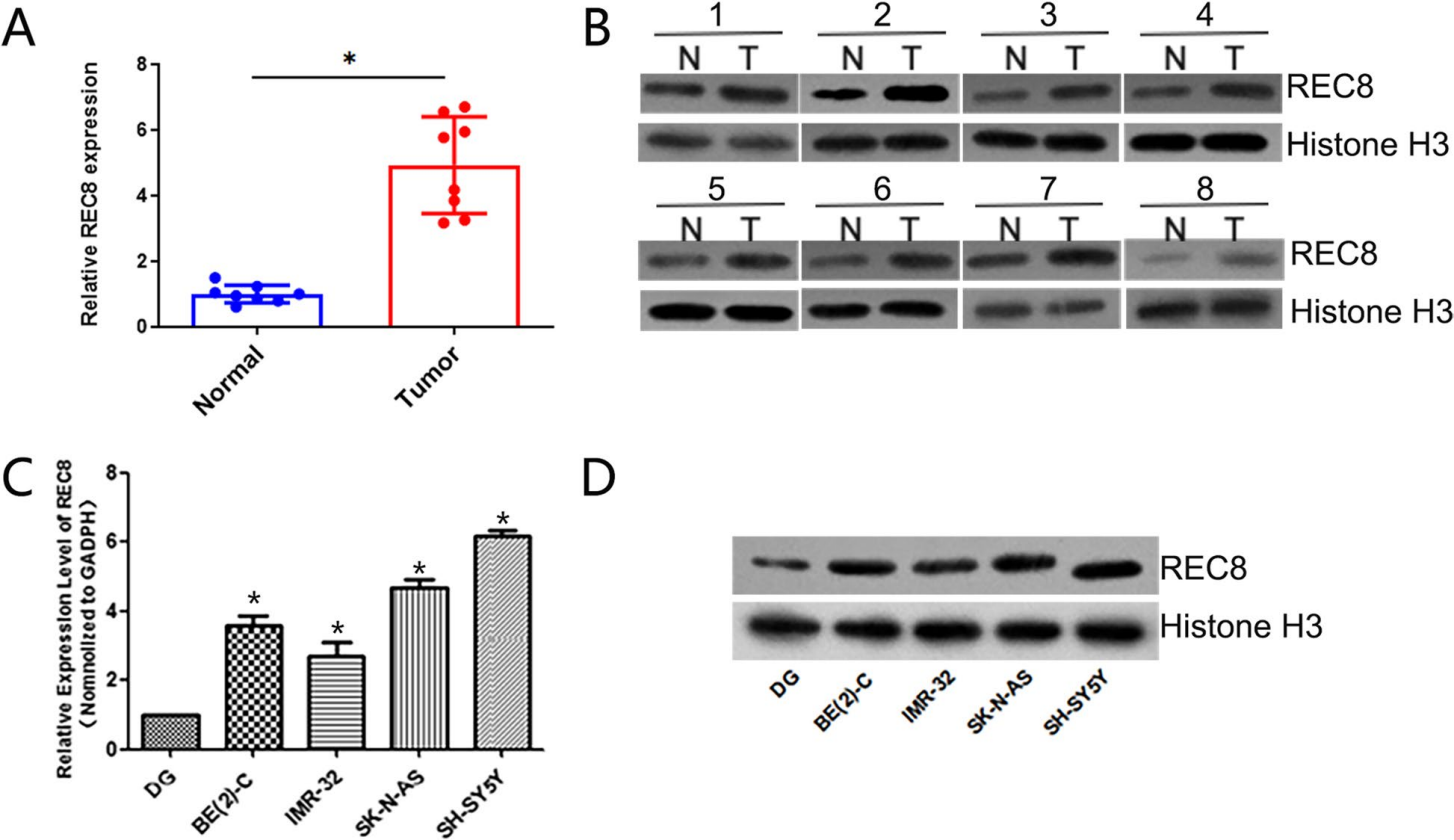
REC8 is upregulated in NB tissues and cells. **A**, **B** qRT-PCR and Western blot were used to detect the mRNA and protein level of REC8 in 8 cases of neuroblastoma. **C**, **D** qRT-PCR and Western blot were used to detect the mRNA and protein level of REC8 in neuroblastoma cell lines (BE (2)-C, IMR-32, SK-N-AS, and SH-SY5Y). T, tumor, N, normal

The expression level of REC8 was investigated using qRT-PCR and Western blot. As the results indicated, the level of REC8 was significantly reduced in NB tissues (Fig. 1A, B) and cells (Fig. 1C, D).

### Downregulation of REC8 significantly inhibits cell proliferation and induces apoptosis in neuroblastoma in vitro

**Fig. 2:**
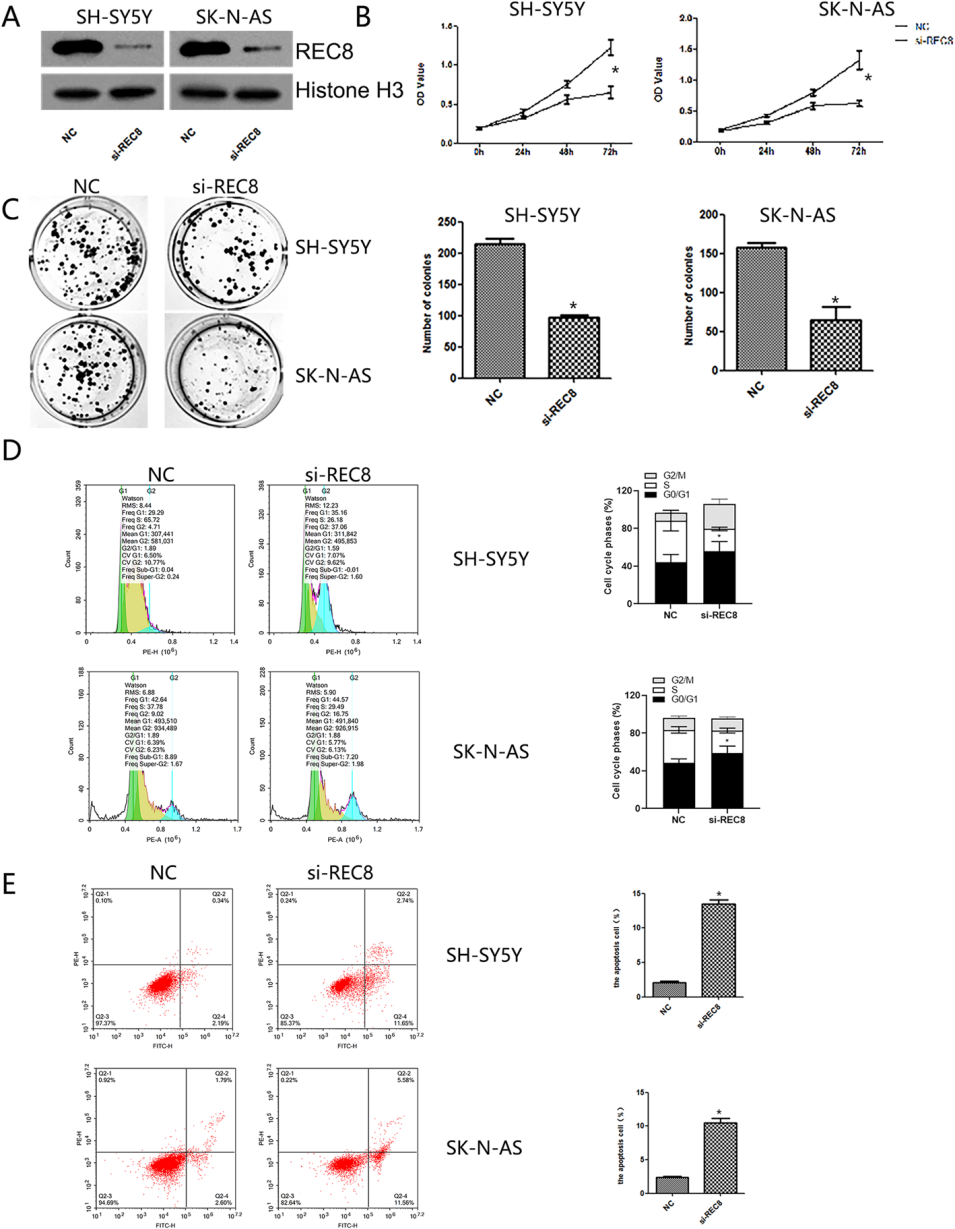
Downregulation of REC8 significantly inhibits cell proliferation and induces apoptosis in neuroblastoma in vitro. **A** Western blotting detected the protein expression of REC8 in NB cells transfected with si-REC8 and negative control. **B** CCK8 was used to evaluate the cell viability of NB cells after REC8 knockdown transfection. **C** Colony formation of NB cells. **D** Flow cytometry was used to analyze the cell cycle of the SH-SY5Y and SK-N-AS cell lines. **E** Flow cytometry detected the cell apoptosis of NB cells

To explore the effect of REC8 on NB progression, we transiently transfected SH-SY5Y and SK-N-AS cells with the controls of siRNA and REC8 siRNA, respectively. As shown in Fig. 2A, protein levels of REC8 were significantly reduced in the si-REC8 group compared with the NC group. Next, we found that REC8 silencing could suppress NB cell viability (Fig. 2B), colony formation (Fig. 2C), and cell cycle G1/S phase transformation (Fig. 2D) and induce cell apoptosis (Fig. 2E).

### Effects of REC8 knockdown on NB cells migration and invasion

**Fig. 3:**
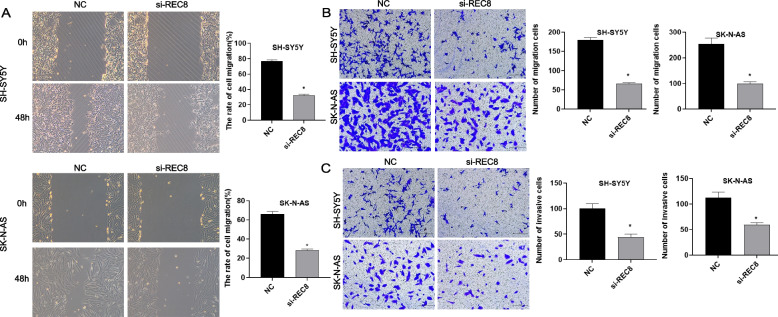
Effects of REC8 knockdown on NB cell migration and invasion. **A** Cell migration was determined using the wound healing migration assay. **B** Migration and **C** invasion of NB cells transfected with si-REC8 measured by Transwell assay

According to the wound healing assay and Transwell assay, the downregulation of REC8 reduced the migration and invasion of SH-SY5Y and SK-N-AS cells (Fig. 3A–C).

### Silencing the REC8 gene by siRNA inhibits NB cells' angiogenesis

**Fig. 4:**
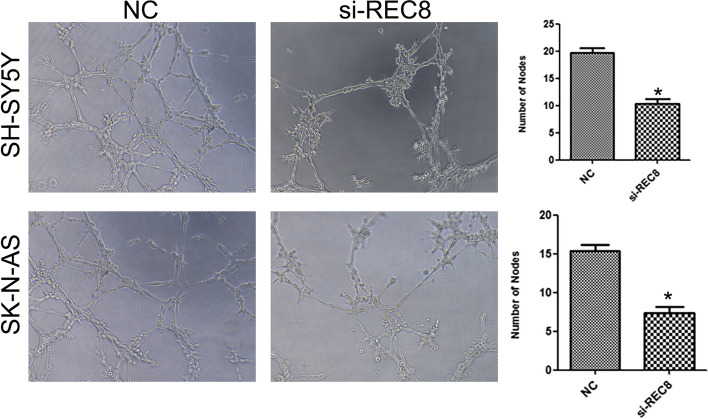
Silencing the REC8 gene by siRNA inhibits NB cell angiogenesis

To explore the possible role of REC8 in tumor angiogenesis, we transiently transfected SH-SY5Y and SK-N-AS cells with the controls of siRNA and REC8 siRNA, respectively. As shown in Fig. 4, the tube-forming assay showed that the downregulation of REC8 inhibited the angiogenesis of NB cells.

### Downregulation of REC8 significantly inhibits STAT3/VEGF Signaling in NB cells

**Fig. 5:**
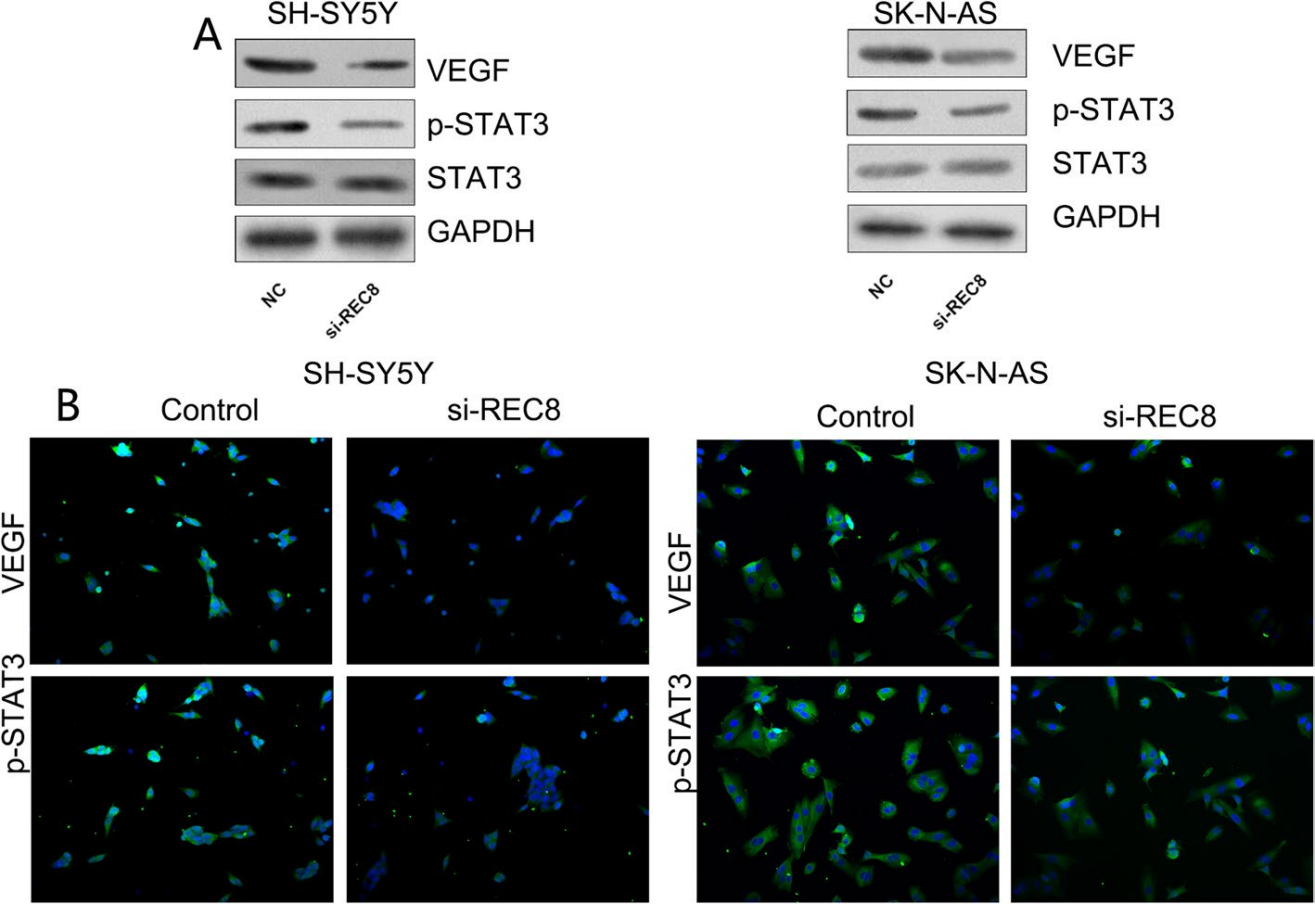
Downregulation of REC8 significantly inhibits STAT3/VEGF Signaling in NB cells. **A** Western blot was used to investigate the VEGF, STAT3, and p-STAT3 proteins in NB cells. **B** Immunofluorescence assays were performed to assess protein expression

Additionally, the potential molecular mechanisms underlying these activities of REC8 were also explored by determining the expression levels of key proteins in the STAT3/VEGF signaling pathway were evaluated. In the western blot and immunofluorescence assay of SH-SY5Y and SK-N-AS cells, VEGF and p-STAT3 expressions decreased when REC8 was inhibited (Fig. 5A, B), indicating that REC8 participated in the regulation of STAT3/VEGF signal pathway.

## Discussion

Sister chromatid cohesion, homologous chromosome pairing, crossover recombination, and chromosome synapsis are mediated by REC8, a key component of the meiotic prophase chromosomal axis [8, 15]. Our results provide direct evidence that REC8 downregulation suppresses NB cell proliferation, motility, and angiogenesis, supporting our hypothesis that REC8 functions as a tumor-promoting gene. On the other hand, we discovered that REC8 mediated NB development through the STAT3/VEGF pathway.

Despite extensive research into neuroblastoma's oncogenesis, cell growth, proliferation, invasion, and migration [16, 17], the underlying molecular pathways leading to neuroblastoma pathogenesis, recurrence, and metastasis remain poorly known. Higher REC8 expression is inversely related to tumor initiation, progression, and metastasis [18, 19]. When we reduced REC8 expression, cell viability and proliferation were drastically reduced. We also discovered that a significant rise in the percentage of cells in the G0/G1 phase ensued when REC8 was silenced. We also demonstrated in vitro that REC8 sped up NB cell migration and invasion. Significant findings showed a favorable association between REC8 and cancer development, but there was also evidence revealing an adverse effect for REC8 in carcinogenesis. Recent evidence suggests that REC8 is epigenetically downregulated in gastric cancer [13]. The growth and colony formation of thyroid cancer cells is slowed by overexpression of REC8 in previous research [10]. Additionally, it has been demonstrated that REC8 inhibits gastric cancer cell angiogenesis by downregulating vascular endothelial growth factor expression [13]. Furthermore, it was shown that the PI3K pathway targets REC8 in thyroid cancer, and REC8 is a tumor-suppressing gene in this disease [10]. Discrepancies in the roles attributed to REC8 among cancer types may result from the fact that different forms of cancer have been studied in isolation.

The regulatory effects of REC8 on tumorigenesis and tumor progression were studied by examining STAT3/VEGF. Western blotting studies showed that REC8 can effectively down-regulate the expression of p-STAT3 and VEGF in SH-SY5Y and SK-N-AS cell lines. It has been reported that inhibition of the STAT3 pathway decreases VEGF expression and plays a significant role in NB [20] and in the genesis and progression of numerous other tumors [21, 22]. In addition, STAT3 was thought to be the primary transcription factor for the VEGF promoter [23]. STAT3 stimulated VEGF expression by binding to its promoter, and previous research indicated that CD24 regulated STAT3 activity and translocation in colorectal cancer [24].

## Conclusion

In conclusion, our results demonstrated that blocking REC8 in neuroblastoma significantly reduced neuroblastoma cell proliferation, migration, invasion, angiogenesis, induced cell cycle arrest, and enhanced apoptosis through the STAT3/VEGF pathway. In this respect, REC8 is a potential target in the search for new therapeutics for NB.

## Data Availability

Data and materials supporting the results are available from the corresponding author on reasonable request.

## Abbreviations

NB: Neuroblastoma
QRT-PCR: Quantitative real-time polymerase chain reaction
REC8: Meiotic recombination protein REC8 homolog
CCK-8: Cell Counting Kit-8
